# A new species in the tree genus *Polyceratocarpus* (Annonaceae) from the Udzungwa Mountains of Tanzania

**DOI:** 10.3897/phytokeys.63.6262

**Published:** 2016-06-01

**Authors:** Andrew R. Marshall, Thomas L.P. Couvreur, Abigail L. Summers, Nicolas J. Deere, W.R. Quentin Luke, Henry J. Ndangalasi, Sue Sparrow, David M. Johnson

**Affiliations:** 1 CIRCLE, Environment Department, University of York, York, UK; 2 Flamingo Land Ltd., Kirby Misperton, North Yorkshire, UK; 3 Biology Department, University of York, York, UK; 4 Institut de Recherche pour le Développement, UMR-DIADE, BP 64501, F-34394 Montpellier cedex 5, France; 5 Université de Yaoundé I, Ecole Normale Supérieure, Département des Sciences Biologiques, Laboratoire de Botanique systématique et d’Ecologie, B.P. 047, Yaoundé, Cameroon; 6 Netherlands Centre for Biodiversity Naturalis (section NHN), Biosystematics Group, Wageningen University, the Netherlands; 7 Durrell Institute of Conservation and Ecology, School of Anthropology and Conservation, University of Kent, Canterbury, UK; 8 East African Herbarium, National Museums of Kenya, Nairobi, Kenya; 9 Department of Botany, University of Dar es Salaam, Dar es Salaam, Tanzania; 10 Department of Botany & Microbiology, Ohio Wesleyan University, Delaware, Ohio 43015 USA

**Keywords:** East Africa, Eastern Arc, endemism, Ndundulu, Polyceratocarpus

## Abstract

*Polyceratocarpus askhambryan-iringae*, an endemic tree species of Annonaceae from the Udzungwa Mountains of Tanzania, is described and illustrated. The new species is identified as a member of the genus *Polyceratocarpus* by the combination of staminate and bisexual flowers, axillary inflorescences, subequal outer and inner petals, and multi-seeded monocarps with pitted seeds. From *Polyceratocarpus scheffleri*, with which it has previously been confused, it differs in the longer pedicels, smaller and thinner petals, shorter bracts, and by generally smaller, less curved monocarps that have a clear stipe and usually have fewer seeds. Because *Polyceratocarpus askhambryan-iringae* has a restricted extent of occurrence, area of occupancy, and ongoing degradation of its forest habitat, we recommend classification of it as Endangered (EN) on the IUCN Red List.

Endangered

## Introduction

The Eastern Arc Mountains of Kenya and Tanzania are well known for their high levels of biodiversity and endemism across many plant and animal groups (Lovett and Wasser 1993, Myers et al. 2000, Newmark 2002, Burgess et al. 2007, Dawson and Gereau 2010). The 13 mountain blocs making up the chain are somewhat isolated from one another, and each block exhibits its own unique suite of species. Many of the forests occupying these mountains are under threat from expanding agriculture and human population increase: an estimated 2.79 Mha of forest was lost between 1908 and 2000 (Willcock et al. 2016), with just 10% of the Eastern Arc Mountains still forested (Platts et al. 2011). These development pressures have added urgency to the inventory of the unique biota of this region.

Within the Eastern Arc chain, the 19,375 km^2^ Udzungwa Mountains form the largest mountain bloc (Platts et al. 2011) and comprise the largest area of forest in the Eastern Arc Mountains, totaling around 1,600 km^2^ (Marshall et al. 2010). The Udzungwa Mountains have enormous biodiversity value, with the highest plant species richness, the highest endemic vertebrate species richness, and the second highest endemic plant species richness of all mountain blocs in the region (Platts et al. 2010; Rovero et al. 2014). The distinctive character of Udzungwa biodiversity has been recently highlighted by high-profile mammal species discoveries including the kipunji monkey (*Rungwecebus kipunji*; Davenport et al. 2006) and Udzungwa elephant shrew (*Rhynchocyon udzungwensis*; Rovero et al. 2008).

Species of the flowering plant family Annonaceae are prominent among the understory trees and woody climbers of the Eastern Arc Mountains. In these mountains, there are 50 known Annonaceae species, 16 of which are endemic, including 12 out of 127 reported endemic tree species (R.E. Gereau, unpubl. data).

The genus *Polyceratocarpus* Engl. & Diels (Annonaceae subfamily Malmeoideae, tribe Piptostigmateae, Chatrou et al. 2012) is distinguishable from other African genera of the family by the combination of a tree habit, percurrent tertiary veins of the leaves, axillary (sometimes cauliflorous) inflorescences, occurrence of both staminate and bisexual flowers, petals of the outer and inner whorls roughly equal in size and shape, numerous monocarps with multiple seeds arranged in a single row, and pitted seeds with spiniform ruminations (Couvreur et al. 2009, Couvreur et al. 2012). Engler and Diels (1900) published the genus based on *Polyceratocarpus scheffleri* Engler & Diels, collected in the Usambara Mountains. Since that time seven additional species have been added to the genus, all from western and central Africa.

Over the last 30 years a number of *Polyceratocarpus* specimens have been collected from the Udzungwa Mountains to the south and west of the range of *Polyceratocarpus scheffleri*. It has become clear that these specimens differ consistently from *Polyceratocarpus scheffleri* and other congeners by a combination of vegetative, floral, and fruit characters, and they are described here as a new species.

## Taxonomic treatment

### Polyceratocarpus askhambryan-iringae

Taxon classificationPlantaeMagnolialesAnnonaceae

A.R. Marshall & D.M. Johnson sp. nov.

urn:lsid:ipni.org:names:77155232-1

Figs 1 , 2 , 3

#### Diagnosis.

This species may be distinguished from other species of *Polyceratocarpus* by the combination of glabrous non-glaucous leaves with finely reticulate to weakly scalariform tertiary venation, pedicels 15–22 mm long, broadly ovoid buds, chartaceous petals 10–17 mm long, 5 to 18 carpels/monocarps, and relatively large torulose monocarps.

#### Type.

*Marshall 2117* (holotype K; isotypes DSM, MO, NHT), Tanzania, Iringa Region: Ndundulu Forest, Kilombero Nature Reserve, Udzungwa Mountains, 07°48'S, 36°31'E (WGS84), 1490 m, 30 May 2011.

#### Description.

Monopodial *tree* to 20 m tall, 4.0–25.4 cm diam.; bark smooth, sparsely lenticellate, often with weak horizontal striations and pits on large trees, grey-brown; branches spirally arranged on trunk, branching from half to two fifths of the height of the main stem, perpendicular but sinuous and drooping slightly; twigs longitudinally rugulose, inconsistently marked with small but prominent lenticels, glabrous, brown. *Leaves*: petiole 4–9 mm long, 1.6–3.3 mm thick, roughened, black, glabrous; lamina narrowly to broadly elliptic-oblong to oblanceolate, or rarely obovate, (5.0-)9.0–25.7 by (3.7-)4.9–8.6(-11.6) cm, chartaceous to coriaceous, greenish gray in sicco, glabrous on both surfaces, base rounded and minutely subcordate, apex acuminate with the acumen 12–20 mm long or occasionally obtuse, midvein plane to slightly impressed above, raised below, secondary veins 9 to 17 per side, diverging at 45–60° from midrib, eucamptodromous to weakly brochidodromous, slightly raised to slightly impressed above, raised below, tertiary veins finely reticulate to somewhat scalariform, indistinct to slightly raised above, raised and conspicuous below. *Inflorescences* 1- or 2-flowered, axillary or occasionally ramiflorous, forming tubercles on leafless growth; pedicels 15–29 mm long 1–3 mm diam., finely appressed-puberulent, bearing a bract 0.8–1 mm long 1/4–2/5 of the distance above pedicel base. Flowers bisexual or staminate, buds broadly ovoid; sepals 3, valvate, crescent-shaped, 2–3.5 mm long, partially connate at the base so that as corolla expands the calyx becomes discoid to triangular with diameter of 7–8 mm, appressed-puberulent abaxially; petals in two whorls of 3, pale yellow in vivo; outer petals occasionally tinged pink on abaxial surface in vivo, valvate, spreading horizontally and recurving at anthesis, narrowly elliptic to elliptic or oblong-elliptic, 10–16 mm long by (5-)8–11 mm wide, coriaceous, apex obtuse, sparsely pubescent adaxially, ferruginous appressed-puberulent but becoming glabrate and verrucose abaxially; inner petals sometimes with a pale brownish-yellow median stripe abaxially, valvate, erect at anthesis with the apices recurved, narrowly elliptic-lanceolate, elliptic, or elliptic-oblanceolate, 11–17 mm long × 5–7 mm wide, coriaceous, external surface marked by a broad flattened ridge that narrows from base to apex, glabrous adaxially, appressed-puberulent with trichomes densest along ridge and at apex abaxially, verrucose on both surfaces, apex acute; stamens ca. 200, 2.0–2.8 mm long, clavate, apex of connective obliquely truncate, pale brown with orange apex, glabrous?; carpels 5 to 18, oblong, 2.9–4.0(-6.0) mm long by 0.9–1.1(-2.3) mm wide, densely pale brown/ferruginous-puberulous; stigma bilobed, capitate, 1 mm in diam., glabrous, ovules ca. 10, uniseriate; torus subglobose to broadly pyriform to oblate, 4.4–4.9 mm long by 2.6–5.4 mm diam., 3–8 mm diam. at base. Pedicel of fruit 20–44 mm long by 3–7 mm diam., weakly longitudinally rugulose, glabrate; torus of fruit ellipsoid to broadly pyriform, 7–15 mm diam. × 8–12 mm long, grey-brown. *Monocarps* up to 18 per fruit, green (rarely with orange or vinaceous tinge) in vivo, dark brown when dried, weakly (to strongly) recurved-falciform, (1.9) 6.0–8.6 cm by 0.7–2.2 cm, torulose, minutely verrucose, glabrate or with a few scattered hairs, base sub-sessile or short-stipitate, stipe 1–11 mm long, 2–6 mm thick, apex rounded or sometimes short-beaked. *Seeds* 1–15 per monocarp, 15 mm long by 13 mm wide by 10 mm thick, arranged in a single [or two irregular?] rows, flattened-ellipsoid, pitted, with spiniform ruminations (fig. 3) and raphe/antiraphe sunken in a circumferential groove.

#### Distribution.

**Figure 1.**
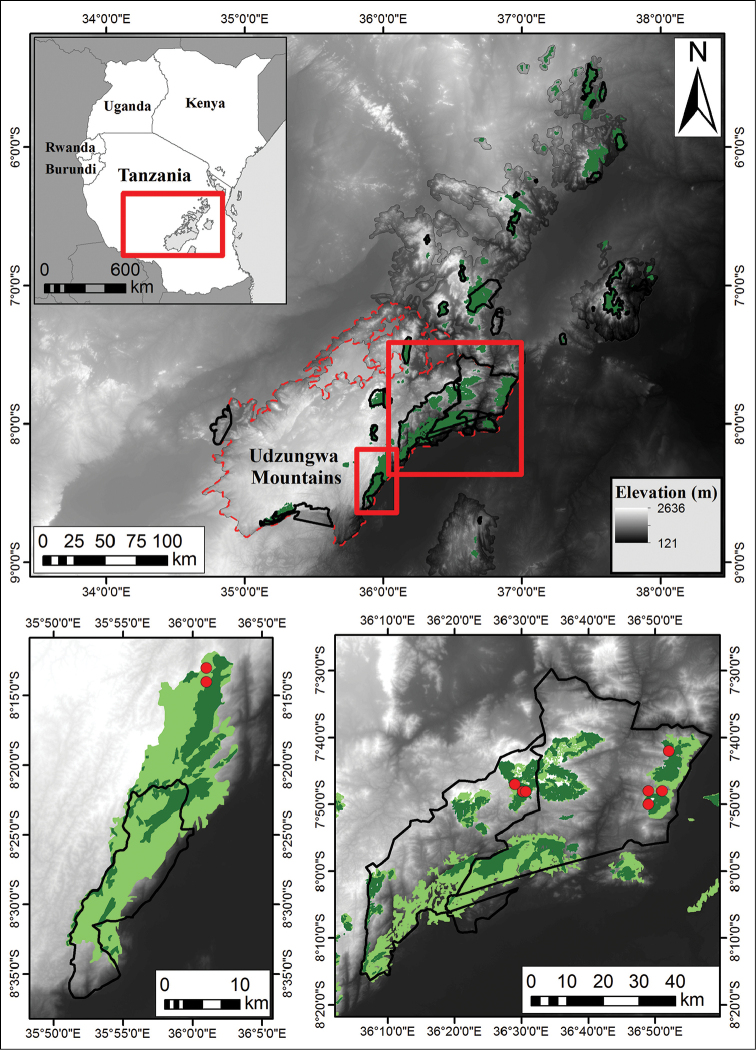
Known distribution of *Polyceratocarpus askhambryan-iringae* in the Eastern Arc Mountains (EAM). EAM boundary and forest cover (green) derived from Platts et al. (2011). Black boundaries within EAM boundary show protected areas. Pale green areas in the lower maps show degraded forest with canopy <10%.

*Polyceratocarpus askhambryan-iringae* is endemic to the Udzungwa Mountains of Tanzania. It is known from Mwanihana Forest in the Udzungwa Mountains National Park, Ndundulu Forest in the Kilombero Nature Reserve, and the Uzungwa Scarp Forest Reserve (Fig. 1).

**Figure 2.**
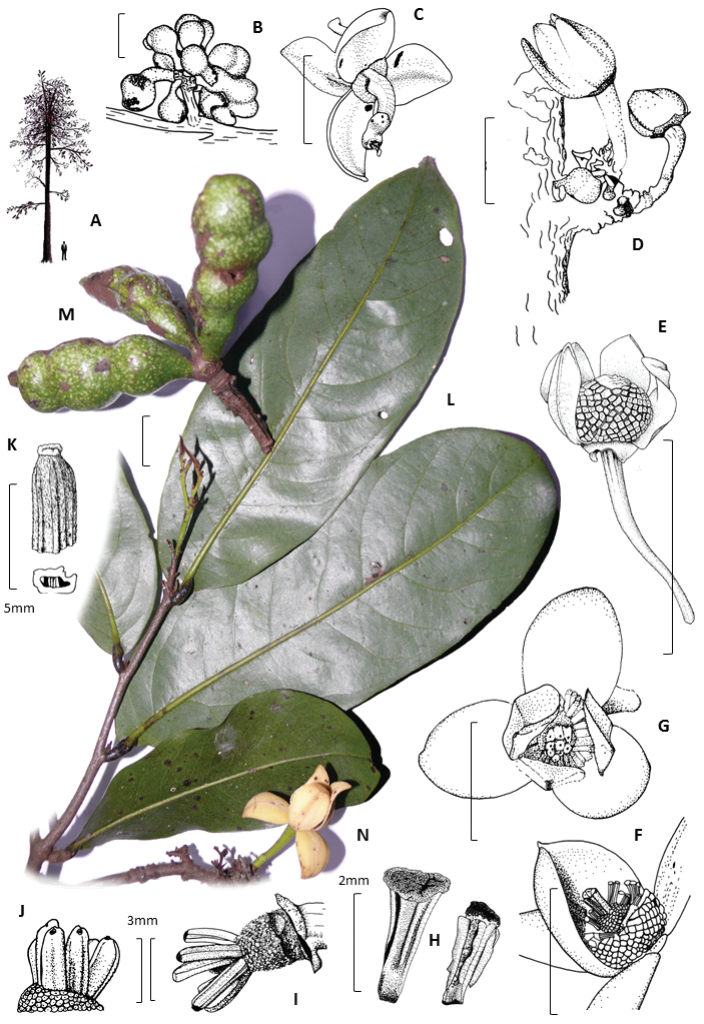
*Polyceratocarpus askhambryan-iringae* drawings of **A** tree architecture **B** fresh fruits **C** fresh flower below **D** fresh ramiflorous flower buds **E–F** dry and fresh bisexual flower (one petal removed) **G** fresh bisexual flower above **H** dried stamens **I**–**J** fresh and dry carpels lacking stigmas **K** dried carpel with stigma, plus photographs of **L** fresh leaves **M** fruit and **N** flower. Drawings by Sue Sparrow, **A** by Andrew Marshall, **E** and **K** by Andrew Brown, from the following specimens: Marshall 2070 (**B**); Marshall 2117 (**C-E** and **G-L**) and Luke 11279 (**F**). Scale bars: 20 mm unless stated.

**Figure 3.**
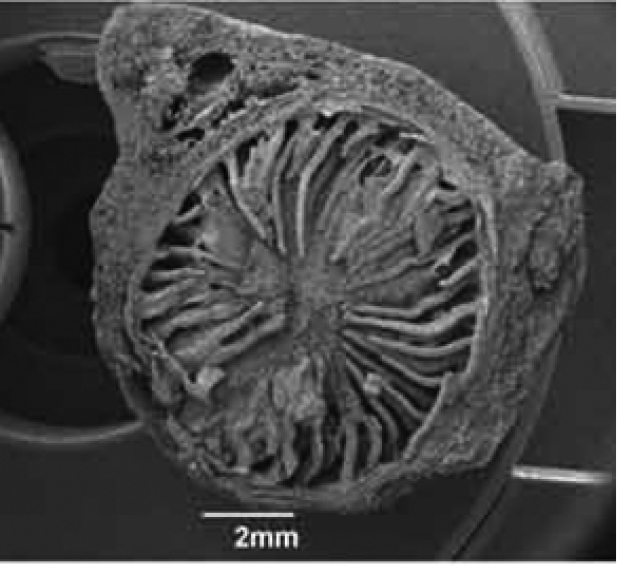
SEM photograph of *Polyceratocarpus askhambryan-iringae* dried seed cross-section, showing spiniform ruminations.

#### Habitat and ecology.

Inhabits montane forest on brown sandy loam soils (pH range 4–5 measured in Ndundulu Forest). Mean annual rainfall of collection localities approximately 1500–2000 mm/yr (Marshall, Ndangalasi, unpubl. data). Thirty-eight mature individuals were found mostly on slopes or ridge-tops at elevations 1090–1540m. Mature flowers were collected in May, November, and December, fruits in February and May-October.

Associated taxa recorded with *Polyceratocarpus askhambryan-iringae* include the following: **(1) Ndundulu Forest**: *Allanblackia ulugurensis* Engl., *Alsodeiopsis schumannii* Engl.; *Anisotes pubinervius* (T.Anderson) Heine, *Anthocleista grandiflora* Gilg, *Beilschmiedia kweo* (Mildbr.) Robyns & Wilczek, *Bertiera pauloi* Verdc., *Cassipourea gummiflua* Tul.; *Cassipourea malosana* Alston; *Chlorophytum brachystachyum* Baker, *Cleistanthus polystachyus* Hook.f. ex Planch., *Clerodendrum cephalanthum* Oliv., *Coffea* sp., *Cola greenwayi* Brenan, *Cola stelechantha* Brenan; *Craterispermum longipedunculatum* Verdc.; *Diospyros abyssinica* (Hiern) F.White, *Drypetes gerrardii* Hutch.; *Englerina* sp. nov.; *Garcinia buchananii* Baker; *Garcinia volkensii* Engl.; *Grewia mildbraedii* Burret; *Justicia rodgersii* Vollesen; *Lasiodiscus usambarensis* Engl.; *Maytenus undata* (Thunb.) Blakelock; *Monodora globiflora* Couvreur; *Monanthotaxis schweinfurthii* Engl. & Diels; *Myrianthus holstii* Engl., *Ocotea usambarensis* Engl.; *Ochna holstii* Engl.; *Parinari excelsa* Sabine, *Peddiea fischeri* Engl., *Pavetta nitidissima* Bridson, *Plectranthus leptophyllus* (Baker) A.J.Paton, Rinorea angustifolia Baill. subsp. ardisiiflora (Oliv.) Grey-Wilson; *Rinorea* sp.; Rytigynia lichenoxenos (K.Schum.) Robyns subsp. glabrituba Verdc.; *Sclerochiton obtusisepalus* C.B.Clarke; *Solanecio epidendricus* (Mattf.) C.Jeffrey; *Strombosia scheffleri* Engl.; *Strychnos mellodora* S.Moore; *Strychnos mitis* S.Moore; *Strychnos* sp. nov; *Syzygium guineense* DC.; *Tabernaemontana stapfiana* Britten; *Tarenna pavettoides* (Harv.) Sim; *Thalictrum rhynchocarpum* Quart.-Dill. & A.Rich.; *Uvariopsis lovettiana* Couvreur & Q.Luke; *Vepris stolzii* I.Verd.; Vernonia calvoana Engl. subsp. leucocalyx (O.Hoffm.) C.Jeffrey; *Vernonia luhomeroensis* Q.Luke & Beentje; Vernonia sp. nr. pteropoda Oliv. & Hiern; *Warneckea* sp. nov.; *Xymalos monospora* Baill.; *Zanthoxylum paracanthum* (Mildbr.) Kokwaro; Zehneria sp. nr. oligosperma C.Jeffrey. **(2) Uzungwa Scarp Forest Reserve**: *Cassipourea gummiflua* Tul; *Cleistanthus polystachyus* Hook.f. ex Planch.; *Craterispermum longipedunculatum* Verdc.; *Diospyros uzungwaensis* Frim.-Møll. & H.J.Ndangalasi; *Drypetes gerrardii* Hutch.; *Lasiodiscus usambarensis* Engl.; *Psychotria megalopus* Verdc.; *Tabernaemontana stapfiana* Britten; *Tarenna uzungwaensis* Bridson. **(3) Mwanihana**: Acalypha psilostachya Hochst. ex A.Rich. var. psilostachya; *Anisotes pubinervius* (T.Anderson) Heine; *Caloncoba welwitschii* Gilg; *Chrysophyllum gorungosanum* Engl.; Coffea mufindiensis Hutch. ex Bridson subsp. mufindiensis; Dorstenia sp. aff tenuiradiata Mildbr.; Isoglossa lactea Lindau ex Engl. subsp. lactea; *Isolona linearis* Couvreur; *Newtonia buchananii* (Baker) G.C.C.Gilbert & Boutique; *Ochna holstii* Engl.; *Parinari excelsa* Sabine; *Phyllopentas ulugurica* (Verdc.) Kårehed & B. Bremer; *Kedrostis* sp.; Polystachya sp aff. canaliculata Summerh.; *Raphidiocystis chrysocoma* (Schumach.) C.Jeffrey; *Selaginella kraussiana* (Kunze) A.Braun; *Stellaria mannii* Hook.f.; *Tricalysia aciculiflora* Robbr.; *Uvariopsis lovettiana* Couvreur & Q.Luke; *Vepris nobilis* (Delile) Mziray; *Zanthoxylum paracanthum* (Mildbr.) Kokwaro.

#### Additional specimens examined.

TANZANIA. Iringa Region, Kilolo District: east Udzungwa National Park, forest south of Mwanihana hill c. 2 km S of last camping site of Mwanihana trail, 1400 m, 07°48'S, 36°49'E, Couvreur 101 (DSM, OWU, WAG); Mwanihana Forest above Sanje village, 1220 m, no grid reference, Lovett 222 (K); Udzungwa Mountains National Park, 1200 m, 07°48'S, 36°49'E, Luke 7738 (EA, K); Udzungwa Mountains National Park, 1440 m, 07°42'S, 36°52'E, Luke 11279 (EA, NHT, MO, K); Ndundulu FR, Camp 589-Camp 590, 07°47'S, 36°29'E, 1440 m, Luke et al. 10366 (MO); Kilombero Nature Reserve, Ndundulu Forest, 1540 m, 07°48'S, 36°31'E (WGS84), Marshall 2036, 2070, (NHT, MO, K); Uzungwa Scarp Forest Reserve, Uluti, 1534 m, 08°14'S, 36°01'E, Ndangalasi HJN 392 (DSM, OWU); Uzungwa Scarp Forest Reserve, Ilutila, 1709 m, 08°13'S, 36°01'E, Ndangalasi 393 (DSM, OWU); Udzungwa, Kilombero FR, W of Ruaha River, 1700 m, Rogers & Hall 2300 (K); Mwanihana Forest above Sanje village, 1400 m, 07°50'S, 36°49'E, Thomas 3656 (MO); Mwanihana Forest above Sanje village, 1400 m, 07°50'S, 36°49'E, Thomas 3698 (MO, WAG). Morogoro Region, Kilombero District: Sonjo-Mwanihana trail, 1090 m, 07°48'S, 36°51'E, Luke 5051 (EA, K).

An additional specimen from Iringa Region (Nyambanitu Forest, Ede 65, K), may also represent this species but bears only an old fruit pedicel lacking monocarps. Further potential *Polyceratocarpus* collections from Iringa Region (Lulanda Forest Reserve: Gereau 2651, 2664, 2665, MO; Lovett 2256, MO, WAG; Luke & Luke 12779, EA & K), were identified as neither *Polyceratocarpus askhambryan-iringae* nor *Polyceratocarpus scheffleri*, while another from Morogoro Region was not considered to be from this genus at all (Kimboza Forest Reserve: Parry 1816, TFD; cited Verdcourt 1971).

#### Additional field notes.

*Slash* dry, slightly stringy, pale yellow (to pale peach), occasionally streaked yellow-brown, dark brown at outer edge formed by the colour of the inner bark, potpourri aroma. *Leaf* lamina dark green above, mid-green with greyish tinge below, turning greenish-grey when dried, new flush pinkish; petiole initially pale green in vivo, becoming roughened grey-brown with age; midrib yellow-green above and below in vivo. *Flower* buds broadly ovoid, green or pale brown-yellow with occasional pink tinge at apex in vivo; sepals yellowish-green in vivo.

#### Etymology.

This new species of *Polyceratocarpus* was named by Askham Bryan College and Iringa International School as part of a rainforest education program.

#### Conservation status.

Our IUCN Red List assessment for *Polyceratocarpus askhambryan-iringae* was based on “area of occupancy” (AOO), “extent of occurrence” (EOO; IUCN 2012) and the level of threat. The 38 observed mature *Polyceratocarpus askhambryan-iringae* stems were found at four collection localities, with only 112 km between the most distant individuals. The four collection localities represented three different levels of governmental protection, including Forest Reserve (Uzungwa Scarp), Nature Reserve (Kilombero, KNR; and also Uzungwa Scarp proposed status) and National Park (Udzungwa Mountains; UMNP), with only UMNP having the maximum level of protection under Tanzanian law (IUCN category II; Dudley 2008). At the time of survey, the greatest threat to *Polyceratocarpus askhambryan-iringae* was widespread timber-felling observed in Uzungwa Scarp Forest Reserve. Conversely, threats to *Polyceratocarpus askhambryan-iringae* in KNR and UMNP were much lower, both comprising only very occasional removal of pole-sized trees by villagers, and potential damage from an increasing elephant population (Marshall et al. 2012). KNR was further threatened by a lack of formal ranger patrols and rapid population expansion in the nearest villages. Given that the sources of threat were closely associated with the three protected areas, for the purpose of Red List assessment we considered three rather than four threat-defined “locations” (IUCN 2012). In calculating AOO, we used 10 km^2^ grid cells so that the projected area was not extrapolated far beyond the expected habitat tolerance of the species. We estimated an AOO of 300 km^2^ and an EOO of 1,410 km^2^, with EOO, AOO, habitat quality and the number of mature individuals, all presumed declining as a result of timber-felling in USFR. Given this continuing decline, plus an EOO of less than 5,000 km^2^, an AOO of less than 500 km^2^, and a population at no more than five locations, the species qualified firmly as endangered on the IUCN Red List (IUCN 2012), EN B1ab(i,ii,iii,v)+2ab(i,ii,iii,v). Within the Udzungwa Mountains, closed-canopy forest was not extensive at elevations suitable for *Polyceratocarpus askhambryan-iringae*, and hence we expect that future expeditions will not expand the AOO or EOO of *Polyceratocarpus askhambryan-iringae* above the IUCN endangered threshold. However, the Rubeho and Mahenge mountain blocs adjacent to Udzungwa were more poorly known, and may contain suitable habitat in which this species might also be found.

## Discussion

### A) Systematics

*Polyceratocarpus askhambryan-iringae* is similar to *Polyceratocarpus scheffleri* and has previously been confused with it. As far as is known, however, *Polyceratocarpus scheffleri* is confined to the Usambara Mountains and *Polyceratocarpus askhambryan-iringae* to the Udzungwa Mountains; previous reports of *Polyceratocarpus scheffleri* from the Udzungwa Mountains (e. g. Lovett et al. 1988, Couvreur et al. 2006, Eastern Arc Mountains & Coastal Forests CEPF Plant Assessment Project 2009) have been based on misidentifications of *Polyceratocarpus askhambryan-iringae*. To facilitate separation of the two East African species, their differences are contrasted in the following key:

**Table:** 

1	Lamina rounded and minutely subcordate at base, with tertiary veins reticulate to weakly scalariform; pedicel 15–29 mm long, bearing a bract 0.8–1 mm long; sepals connate only at base, distinct, crescent-shaped; petals 10–17 mm long; monocarps up to 18 per fruit, (1.9–) 6–8.6 cm long, weakly recurved-falciform, short-stipitate (1–11 × 2–6 mm); seeds per monocarp up to 15	***Polyceratocarpus askhambryan-iringae***
–	Lamina cuneate to broadly cuneate at base, with tertiary veins distinctly scalariform; pedicel 10–16 mm long, bearing a bract 7–9 mm long; sepals connate into a disc with indistinguishable sepal lobes; petals 18–32 mm long; monocarps 5 to 33, 6–20 cm long, strongly curved, sessile; seeds per monocarp up to ca. 25	***Polyceratocarpus scheffleri***

The genus *Polyceratocarpus* outside East Africa has a Guineo-Congolean distribution, with the other seven species scattered from Côte d’Ivoire to northern Angola and the Democratic Republic of the Congo. *Polyceratocarpus askhambryan-iringae* and *Polyceratocarpus scheffleri* both differ from most of the more western species in having larger numbers of carpels and large strongly torulose monocarps. The only other species with such a high number of carpels (18 to 20) is *Polyceratocarpus laurifolius* Paiva from northern Angola, but that species differs from *Polyceratocarpus askhambryan-iringae* in having densely pubescent twigs, a distinctly cuneate base to the leaf, a larger bract on the pedicel, and larger sepals. *Polyceratocarpus laurifolius* also differs from *Polyceratocarpus askhambryan-iringae* in having larger petals of the staminate flowers than of the bisexual ones (Paiva 1966); in *Polyceratocarpus askhambryan-iringae* no petal dimorphism between staminate and bisexual flowers was seen.

*Polyceratocarpus askhambryan-iringae* (as “*Polyceratocarpus* sp.”) was one of four *Polyceratocarpus* species included in the phylogenetic analysis of Couvreur et al. (2009) focused on the phylogeny of several closely related African genera of Annonaceae. In this analysis *Polyceratocarpus askhambryan-iringae* appeared as sister to the other three species of *Polyceratocarpus* sampled (*Polyceratocarpus microtrichus* (Engl. & Diels) Ghesq. & Pellegr., *Polyceratocarpus parviflorus* (Baker f.) Ghesq., and *Polyceratocarpus pellegrinii* Le Thomas) forming a monophyletic group with strong bootstrap and posterior probability support. The other five species remain to be sampled. The genus itself, however, was nested within the western and central African genus *Piptostigma* Oliv., to which it is morphologically dissimilar, so additional sampling is needed.

### B) Regional endemism and biodiversity

East Africa is an area of both high endemism and high diversity for Annonaceae, with 28 genera and 85 species known from Tanzania alone (Couvreur et al. 2006). In particular, the Eastern Arc Mountains form an area of high species endemism for East African Annonaceae. Furthermore, many Annonaceae genera represented in these mountains have main areas of diversity in the Guineo-Congolean region and are represented in the Eastern Arc Mountains by endemic taxa. In addition to *Polyceratocarpus*, the genera *Annickia* Setten & Maas, *Greenwayodendron* Verdc., *Isolona* Engl., *Monodora* Dunal, *Uvariodendron* (Engl. & Diels) R.E. FR., and *Uvariopsis* Engl. all follow this pattern (Verdcourt 1971, 1986, Couvreur et al. 2006, Couvreur 2009, Couvreur and Luke 2010).

*Polyceratocarpus askhambryan-iringae* also adds to the growing list of species unique to the Udzungwa bloc, including various recently discovered plants (e.g. Luke and Beentje 2003; Knox et al. 2004). There are now 71 known endemic plant species from the Udzungwa Mountains (comprising 15 trees, including *Polyceratocarpus askhambryan-iringae*); among the Eastern Arc Mountains this is second only to the Uluguru Mountains (86 endemic species [14 trees]; R.E. Gereau, unpubl. data). *Polyceratocarpus askhambryan-iringae* is the second large tree (≥20m) and the third endemic Annonaceae species to be described from the Udzungwa Mountains over the last 20 years, following the respective discoveries of *Omphalocarpum strombocarpum* Y.B.Harv. & Lovett (Harvey and Lovett 1998), *Toussaintia patriciae* Q.Luke & Deroin (Deroin and Luke 2005) and *Monodora globiflora* Couvreur (Couvreur et al. 2006). Given further new species descriptions in preparation and the larger size of the Udzungwa Mountains, it is likely to be the most important Eastern Arc Mountain bloc for tree species endemism. More plant species are known from the Udzungwa Mountains than other Eastern Arc Mountain blocs, and while this is partly due to increased survey effort over other blocs, recent projections by distribution models estimate that the number of endemic/threatened plant taxa will not be superseded following increased exploration of other blocs (Platts et al. 2010).

The four *Polyceratocarpus askhambryan-iringae* localities are coextensive with other recent discoveries of rare and endemic Eastern Arc animals, e.g. the Critically Endangered endemic Sanje mangabey (*Cercocebus sanjei*; Mwanihana and Uzungwa Scarp), the Critically Endangered kipunji monkey (*Rungwecebus kipunji*; Ndundulu; also found on Rungwe Mountain), the endemic Udzungwa forest partridge (*Xenoperdix udzungwensis*; Ndundulu), the endemic rufous-winged sunbird (*Nectarinia rufipennis*; Mwanihana, Ndundulu and Uzungwa Scarp), the endemic Udzungwa elephant shrew (*Rhynchocyon udzungwensis*; Ndundulu and Mwanihana), further emphasizing the exceptional local biodiversity value. The exceptional biodiversity of these areas led to the incorporation of Mwanihana forest into the Udzungwa Mountains National Park in 1991, and later incorporation of Ndundulu forest into the Kilombero Nature Reserve in 2007 (Marshall et al. 2007).

The discovery of *Polyceratocarpus askhambryan-iringae* further highlights the need for improved conservation of Uzungwa Scarp Forest Reserve, one of the most important forests in the region for primates and birds (Dinesen et al. 2001), where herpetofauna endemism has been estimated at eight times that of the Eastern Arc as a whole (Menegon, unpublished data). Uzungwa Scarp FR is home to the Kihansi spray toad (*Nectophrynoides asperginis*), which was extinct in the wild, but was successfully reintroduced in October 2012 following zoo conservation breeding (Channing et al. 2009; Gereau et al. 2014). We hope that our discovery of yet another new endemic will add weight to ongoing proposals for gazettement of Uzungwa Scarp Forest Reserve as a Nature Reserve, the highest designation of protected area possible under the Tanzania Forestry Service.

## Supplementary Material

XML Treatment for Polyceratocarpus askhambryan-iringae
